# Systemic Lupus Erythematosus Presenting as Thrombotic Thrombocytopenia Purpura: How Close Is Close Enough?

**DOI:** 10.1155/2011/267508

**Published:** 2011-05-10

**Authors:** Cesar A. Perez, Nabil Abdo, Anuj Shrestha, Edgardo S. Santos

**Affiliations:** ^1^Division of Hematology/Oncology, Sylvester Comprehensive Cancer Center, University of Miami Miller School of Medicine, 1475 NW 12th Avenue, D8-4 (Suite 3510), Miami, FL 33136, USA; ^2^Rosalind Franklin University of Medicine and Science, 3333 Green Bay Road, North Chicago, IL 60064, USA

## Abstract

Thrombotic thrombocytopenic purpura (TTP) is an uncommon life-threatening disease characterized by microangiopathic hemolytic anemia and thrombocytopenia, commonly associated with infections, malignancy, drugs, and autoimmune diseases. We report a case of 19-year-old previously healthy female that presents with anemia and thrombocytopenia diagnosed with thrombotic thrombocytopenic purpura that was treated successfully with plasmapheresis and corticosteroids. Laboratory findings also revealed antinuclear antibodies and antibodies to double-stranded DNA. Two weeks after presentation developed inflammatory arthritis, fulfilling diagnostic criteria for systemic lupus erythematosus (SLE). Prompt diagnosis and treatment with plasma exchange and corticosteroids should be instituted as soon as the diagnosis of TTP is suspected, even if other diagnoses, including lupus, are possible. When present, the coexistence of these two etiologies can have a higher mortality than either disease alone. An underlying diagnosis of SLE should be considered in all patients presenting TTP and the study of this association may provide a better understanding of their immune-mediated pathophysiology.

## 1. Background

The relation between the systemic lupus erythematous (SLE) and thrombotic microangiopathies, including malignant hypertension, antiphospholipid syndrome, and thrombotic thrombocytopenic purpura (TTP) has always presented several diagnostic difficulties. TTP, an uncommon life-threatening disease, can present in approximately 2% of the patients with SLE [1]. However, this occurs commonly in patients with active SLE disease. TTP as the initial manifestation of SLE is not only an extremely uncommon scenario, but also one with potentially high mortality [1]. Therefore, the presentation of a patient with TTP rarely prompts clinicians to work up for secondary autoimmune diseases, including SLE. Herein, we review the pathophysiology of this relationship and possible new therapeutic approaches for this uncommon clinical scenario. 

## 2. Case Presentation

**Figure 1:**
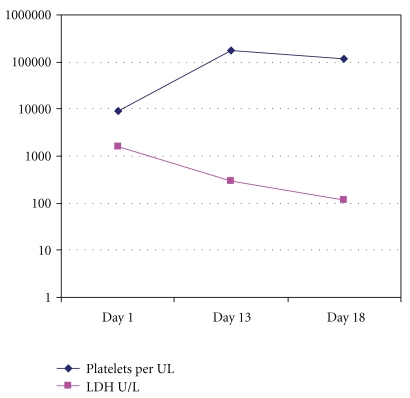
Platelets and LDH logarithmic trend in hospital.

A previously healthy 19-year-old African American female presented to the emergency room complaining of a 2-day history of generalized fatigue, bruising over the upper and lower extremities as well as headaches, but no signs of bleeding from orifices. She did not have any significant past medical history or surgical history. Other than medroxyprogesterone acetate injections for contraception and ibuprofen for infrequent migraine, she was not taking any medications. Her menstrual periods were regular and predictable and she denied any prior pregnancy or abortion. The review of systems was only positive for recent increased hair loss, but otherwise negative. On physical examination she had temperature of 99.1 degree Fahrenheit, pulse was rapid and regular at 116 beats/min, blood pressure of 120/75 mmHg, respiration at 16 per minute. She was fully alert and oriented, but with pale skin and with bruising on upper and lower extremities. Cardiovascular examination revealed only tachycardia but no added sounds, and her abdomen was soft without tenderness or hepatosplenomegaly on palpation. The rest of the physical examination was unremarkable. The initial laboratory workup revealed hemoglobin of 6.1 g/dL, normal MCV, a high reticulocyte index of 4%, thrombocytopenia of 9,000 × 10^−6^/L and normal white blood cell count. Peripheral blood smear revealed 5 schistocytes per hpf, anisocytosis, poikilocytosis, and some tear drop cells as well. The coagulation profile was within the normal limits. The LDH was 1624 IU/L, AST of 90 IU/L, ALT of 78 IU/L, and total bilirubin was 1.9 mg/dL with direct bilirubin of 0.4. Direct Coombs' test was negative, and haptoglobin level was normal, attributed to the inflammatory process that was later revealed. Renal function was preserved with a creatinine of 0.7 mg/dL, and all the serum electrolytes were within the normal range. Urine pregnancy test was negative. Urinalysis revealed proteinuria of 30 mg/dL. She was admitted to the Intensive Care Unit, and treatment was started with methylprednisolone 1 gram intravenously daily for 3 days with a decreased dose thereafter, in conjunction with two daily sessions of plasmapharesis. She improved remarkably, with a normalization of her platelet count in 10 days, and her liver enzymes, LDH, and hemoglobin all were corrected within 2 weeks (Figure 1). 

She also had a positive antinuclear antibody (ANA) screening with speckled pattern and titer of 1 : 640; antidouble stranded DNA (dsDNA) antibodies level was elevated at 36.37 IU/L. Other serologies including lupus anticoagulant, anti-B2-glycoprotein, anticardiolipin, antismith, anti-RNP, C3, and C4 complements levels were all negative. Interestingly, the patient was found to have ADAMTS13 activity below 5% and inhibitor to ADAMTS13 with a level of 0.8 U mL^−1^. One week after finishing the course of plasmapheresis, and after steroids were discontinued, she developed tenderness and swelling of both ankles, fulfilling criteria for SLE diagnosis (hemolytic anemia and thrombocytopenia, inflammatory arthritis, positive ANA and positive dsDNA). Other diagnostic criteria as malar or discoid rash, photosensitivity, oral ulcers, serositis, renal, or neurologic findings were not seen.

## 3. Discussion

The early recognition of TTP in this patient and the prompt workup of her associated underlying disease allowed an early effective treatment and followup, improving not only the immediate outcome, but also her long-term prognosis. 

After the initial description of TTP by Moschowitz in 1924, a pentad of severe thrombocytopenia, microangiopathic hemolytic anemia, neurologic abnormalities, renal insufficiency, and fever was established in 1966 by Amorosi and Ultmann [2, 3]. Since then, the mortality of TTP has decreased dramatically from 90% without treatment to 8–25% with the institution of plasma exchange transfusion [4]. This resulted in the diagnostic criteria to be revised from the earlier pentad to the current dyad of thrombocytopenia and microangiopathic hemolytic anemia, with no clinically apparent alternative explanation for thrombocytopenia and anemia [3, 5–7]. During the last decade, the main pathophysiologic feature of TTP has been described as severe deficiency of von Willebrand Factor (vWF) cleaving metalloproteinase (ADAMTS-13), which normally cleaves the unusually large vWF into smaller and less adhesive vWF moeity. This deficiency is thought to be possibly secondary to the presence of an IgG antibody inhibiting ADAMTS-13 activity, inhibition that finally allows the presence of units of unusually large vWF which is responsible for the microvascular thrombosis, hemolysis, and thrombocytopenia [3, 8].

Connective tissue disorders, including SLE, can present with low levels of ADAMTS-13, suggesting a possible common pathophysiology for these diseases [9]. TTP occurring in patients with SLE can be difficult to diagnose because of overlapping features of the two disorders and the presence of other potentially concomitant thrombotic microangiopathies [9]. When developed in patients with SLE, TTP usually presents in patients previously diagnosed and treated for SLE for several years, with a high SLE disease activity index score and coexisting nephritis, having a higher mortality than either disease alone [1, 9]. The clinical overlap between these two syndromes has been described more commonly in young black women, as the case described [10]. Also, this concomitant scenario has been described to have a “slower tempo” of development, possible because patients diagnosed with SLE were already being treated with immunosuppressive therapy and corticosteroids, a fact that may suppress the immune mechanisms involve in TTP development. However, the delay in establishing the diagnosis of TTP in a patient with SLE might also bias this “slow tempo” of development [11]. 

The inverse sequence of events observed in our case triggers the question of how useful it would be to screen patients that present with suspected TTP with ANA. Although SLE may present with hemolytic anemia, thrombocytopenia, neurologic deficits, fever, and renal insufficiency, the finding of fragmented RBCs or schistocytes favors the diagnosis of TTP [12]. An initial schistocyte count of more than 1% in the absence of any other cause for thrombocytopenia is strongly suggestive of TTP. Although the American College of Rheumatology classification criteria for SLE are >90 percent specific and sensitive, the clinical presentation of isolated TTP may be indistinguishable from that of SLE with secondary TTP during the acute initial episode of TTP [13]. The underlying reason for the association of SLE with TTP is unknown. There is still considerable debate about whether certain antiphospholipid antibodies or lupus anticoagulant play a pathogenic role in triggering TTP in SLE patients, although neither of these two types of antibodies has to be present for SLE patients to develop TTP [14]. The relationship between the ADAMTS-13 inhibitor and SLE also needs to be further investigated, since its presence in patients with SLE and concomitant TTP had not been described in the few small retrospective series available because the test was not available at that time. Mortality rate of TTP in patients with SLE has been reported to be 46–50% in two small series, and was associated with the presence of concomitant infections and the delay in the initiation of plasma exchange therapy [1, 9].

Plasma exchange continues to be the mainstay of treatment in patients with TTP, even when concomitant SLE is present. Other therapies that have been used with variable results include high-dose steroids, cytotoxic agents such as cyclophosphamide and vincristine, and intravenous immunoglobulin. Rituximab, a monoclonal antibody against CD20 receptor, has been used in four reported cases of refractory TTP in SLE patients, with a response and disease-specific survival of 50% [11, 15, 16].

The association of SLE and TTP is uncommon, but potentially lethal, even with current treatment strategies, emphasizing the importance of early diagnosis and aggressive management with plasma exchange and immunosuppression. In refractory cases, the use of rituximab may be an option, although currently its use is limited to a few cases. If diagnosis of either disease is present, the possibility of the other needs to be considered if clinically suggested, since the prognosis and treatment can vary. Critical care specialists, rheumatologists, and hematologists need to evolve an urgent multidisciplinary approach which emphasizes the early recognition of this phenomenon and the initiation of early treatment which secure a better outcome.
